# *In Vivo* Evaluation of the Ameliorating Effects of Small-Volume Resuscitation with Four Different Fluids on Endotoxemia-Induced Kidney Injury

**DOI:** 10.1155/2015/726243

**Published:** 2015-07-26

**Authors:** Yan-ling Wang, Jing-hui Chen, Qiong-fang Zhu, Gao-feng Yu, Chen-fang Luo, Gang-jian Luo, Shang-rong Li, Zi-qing Hei

**Affiliations:** ^1^Department of Anesthesiology, The Third Affiliated Hospital of Sun Yat-sen University, No. 600 Tianhe Road, Tianhe District, Guangzhou 510630, China; ^2^Department of Anesthesiology, Guangzhou Women and Children's Medical Center, No. 9 Jinsui Road, Guangzhou 510623, China; ^3^Department of Anesthesiology, The First Affiliated Hospital of Sun Yat-sen University, No. 58 Zhongshan Road II, Guangzhou 510080, China

## Abstract

Acute kidney injury associated with renal hypoperfusion is a frequent and severe complication during sepsis. Fluid resuscitation is the main therapy. However, heart failure is usually lethal for those patients receiving large volumes of fluids. We compared the effects of small-volume resuscitation using four different treatment regimens, involving saline, hypertonic saline (HTS), hydroxyethyl starch (HES), or hypertonic saline hydroxyethyl starch (HSH), on the kidneys of rats treated with lipopolysaccharide (LPS) to induce endotoxemia. LPS injection caused reduced and progressively deteriorated systemic (arterial blood pressure) and renal hemodynamics (renal blood flow and renal vascular resistance index) over time. This deterioration was accompanied by marked renal functional and pathological injury, as well as an oxidative and inflammatory response, manifesting as increased levels of tumor necrosis factor-*α*, nitric oxide, and malondialdehyde and decreased activity of superoxide dismutase. Small-volume perfusion with saline failed to improve renal and systemic circulation. However, small-volume perfusion with HES and HSH greatly improved the above parameters, while HTS only transiently improved systemic and renal hemodynamics with obvious renal injury. Therefore, single small-volume resuscitation with HES and HSH could be valid therapeutic approaches to ameliorate kidney injury induced by endotoxemia, while HTS transiently delays injury and saline shows no protective effects.

## 1. Introduction

Endotoxemia frequently occurs during critical phases of clinical diseases, including trauma and infectious diseases, and inevitably causes shock and organ damage contributing to poor survival rate [1, 2]. Acute kidney injury (AKI) is a common complication following endotoxic shock and is associated with a high morbidity rate of up to 64% [3]. Despite significant advances in control of endotoxemia, there remains an urgent need to promptly prevent the occurrence of AKI or restore impaired renal function associated with sepsis [4].

In sepsis, renal blood flow is frequently reduced with progressive development of renal inflammation and oxidative stress, thereby contributing to the genesis of AKI [5, 6]. Early resuscitation using crystal or colloid fluids to improve tissue perfusion and inhibit oxidative and inflammatory response may provide a meaningful degree of renal protection [7, 8]. However, infusion of large-volume fluids is always limited to critically ill patients, although caution must be exercised in patients with heart dysfunction, in whom small-volume resuscitation is typically used [9]. The potential protective effects of small-volume fluid resuscitation on kidney injury induced by endotoxemia are currently unknown.

Fortunately, small-volume resuscitation with hypertonic solutions has been successfully applied in clinical rescue of hemorrhagic and septic shock [10–12]. However, the debate persists regarding which solution (crystalloids or colloids) is most beneficial [13, 14]. For instance, it is reported that appropriate administration of colloids seemed to be associated with reduced mortality, while others consider that resuscitation with balanced crystalloids was associated with a lower risk of in-hospital mortality [15, 16]. In the present study, we compared the effects of small-volume resuscitation with saline, 7.5% hypertonic saline (HTS), hydroxyethyl starch 130/0.4 (HES), and hypertonic saline hydroxyethyl starch 40 (HSH) at a dose of 4 mL/kg on acute kidney injury induced by endotoxemia. The potential mechanism of the hypothesized protection was further explored, focusing on renal perfusion and variations of oxidative and inflammatory mediators.

## 2. Methods

### 2.1. Animal Procedures

The study was reviewed and approved by the Research Ethics Committee of The Third Affiliated Hospital of Sun Yat-sen University, Guangzhou, China. Thirty adult male Sprague-Dawley (SD) rats (180–250 g body weight) were provided by the Experimental Animal Center of Sun Yat-sen Medical School and were treated in accordance with both international and institutional guidelines.

Experimental procedures on rats were performed as previously described [10]. Briefly, after anesthesia, animals were ventilated with 40–50% oxygen (Rodent Ventilator 638, Harvard Apparatus, Boston, MA, USA), with proper tidal volume (7 mL/kg) and respiratory rate (approximately 30 breaths/min). The left carotid artery was catheterized for continuous monitoring of arterial pressure. A phased-array, low-frequency probe (frequency 2.0–5.0 MHz) was placed in the left renal door cross-sectional level for kidney color velocity imaging (CVI) detection. All animals were given intravenous administration of 1 mg LPS/kg through the right internal jugular vein to establish the endotoxemia model.

### 2.2. Drug and Fluids

Sodium pentobarbital in saline was obtained from Sigma Chemical Co. (St. Louis, MO, USA). LPS was isolated from* E. coli* 0111:B4 and purchased from Sigma Chemical Co. The experimental solutions were 0.9% saline (Zhejiang Chimin Pharmaceutical Co., Ltd., Taizhou, China), 7.5% hypertonic saline (HTS, The Third Affiliated Hospital of Sun Yat-Sen University, Guangdong, China), hydroxyethyl starch (HES, Fresenius Kabi Pharmaceutical Co., Ltd., Bad Homburg, Germany), and hypertonic saline hydroxyethyl starch (HSH, Shanghai Huayuan Chang Fu Co., Ltd., Shanghai, China).

### 2.3. Experimental Groups

Rats were randomized into five groups, including control and four resuscitation groups (*n* = 6 per group). The groups were designated as follows: (1) group C (control group): 4 mL 0.9% saline/kg; (2) group S (saline group): LPS 1 mg/kg + 4 mL 0.9% saline/kg; (3) group HTS: LPS 1 mg/kg + 4 mL 7.5% HTS/kg; (4) group HES: LPS 1 mg/kg + 4 mL HES/kg; and (5) group HSH: LPS 1 mg/kg + 4 mL HSH/kg. Four kinds of resuscitation solutions at a dose of 4 mL/kg were administered at 30 min after intravenous injection of LPS. And fluids were administered within 5 min according to [17]. After performing systemic hemodynamic and renal blood flow measurements, rats were euthanized, blood was collected by cardiac puncture, and the kidneys were harvested.

### 2.4. Systemic Hemodynamic and Renal Microcirculatory Measurements

Systolic arterial pressure (SAP) was monitored from catheterized internal carotid arteries with a Hewlett-Packard monitor. Renal flow indices were measured by Doppler ultrasound using a Technos MPx DU8 instrument (Esaote Biomedica, Genoa, Italy). Image-Pro Plus Image Analysis software was used to calculate the ratio of renal blood signal and the peak renal cross-section area. SAP and renal microcirculation indices (renal blood flow signals (CVI), peak-systolic velocity (*V*_max⁡_), and end-diastolic velocity (*V*_min⁡_)) were recorded before LPS administration (*T*_0_, baseline), 30 min after LPS administration (*T*_1_), and 10 min (*T*_2_), 30 min (*T*_3_), and 60 min (*T*_4_) after small-volume resuscitation.

Renal vascular resistance index (RVRI) was calculated by the formula

(1) $$\begin{array}{c} \text{RVRI}=\frac{V_{\max}-V_{\min}}{V_{\max}}. \end{array}$$

### 2.5. Renal Function Parameters

Blood samples were obtained from the abdominal aorta 60 min after small-volume resuscitation. Renal function was determined by assaying serum for serum creatinine (Scr) and blood urea nitrogen (BUN) using the AV800 Chemistry Analyzer (Hitachi, Hitachi City, Japan).

### 2.6. Renal Histopathology Images Obtained by Light and Electron Microscopy

Renal tissues were taken 60 min after small-volume resuscitation and fixed in 10% formaldehyde. After being processed in an Autotechnicon, tissues were embedded in paraffin for light microscopy. Sections of 5 *μ*m thickness were cut with a microtome and stained with hematoxylin-eosin (H&E). The degree of each abnormality was graded numerically by the Paller score according to [18].

Separated tissue samples were fixed in 2% paraformaldehyde and 2.5% glutaraldehyde in 0.1 M PBS (pH 7.4) at 4°C for 1 day for electron microscopy. After being postfixed with 2% osmium tetroxide, tissues were dehydrated in graded ethanol and embedded into araldite. The stained sections were examined using a Leo 906E electron microscope.

### 2.7. Detection of Renal Cortical TNF-*α* and NO Levels

Because the renal cortex is highly sensitive to LPS challenge, renal cortical tissue was separated from the rest of the kidney for analyses [19]. Levels of TNF-*α* and NO were quantified with specific ELISA and NO kits (Boster Company, Wuhan, China, and Nanjing Jiancheng Bioengineering Institute, Nanjing, China, resp.).

### 2.8. Detection of Renal Cortical MDA Level and SOD Activity

Renal cortex was homogenized for assessment of MDA level and SOD activity with kits based on the thiobarbituric acid reactive substance assay and xanthine oxidase method, respectively (both by Jiancheng Bioengineering Institute, Nanjing, China).

### 2.9. Data Exclusion and Statistical Analyses

Data were expressed as means ± SEM. Statistical analysis of data was performed by using one-way analysis of variance (ANOVA), while repeated measurements of ANOVA were used for CVI analyses. Tukey's HSD test was used for intragroup comparisons. Pathology scores were analyzed by a nonparametric *t*-test. Differences were considered to be statistically significant if *P* < 0.05.

## 3. Results

### 3.1. Renal Function and Pathology Changes

**Figure 1:**
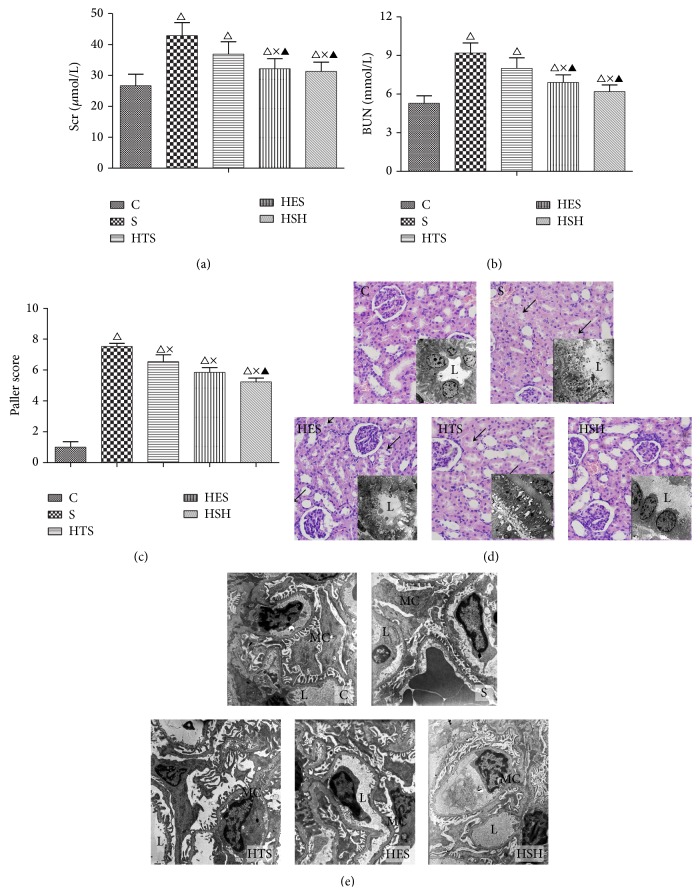
Small-volume resuscitation alleviated the severity of renal injury in rats with LPS-induced endotoxemia. (a) and (b) show alterations in renal function parameters in response to small-volume resuscitation with different solutions after LPS administration. (c) shows Paller scores in different groups. (d) Renal pathology changes under light (stained with HE, ×400) and electron microscopy (for tubular ultrastructure, ×2950). Photomicrograph shows an intact epithelial system with normal renal tubules and glomeruli in group C, while the renal tubule exhibited serious degeneration, disintegration, and edema in epithelial cells as well as cellular debris obstructing the collecting tubules in group S (as shown by ↙); HTS-treated group showed only local epithelial and mitochondrial edema with less necrosis and abscission, while the HES- and HSH-treated groups only showed occasional injury. Ultrastructure (L: lumen, EC: epithelial cells). (e) Ultrastructure of glomerulus under an electron microscope (×5200). Photomicrograph shows normal glomerular structure in group C, while the number of foot processes in podocytes decreased and these foot processes were locally confluent with small amounts of dense deposits inside the basement membrane in group S. However, the podocytes were restored to a normal state with no dense deposits in photomicrographs from the HTS-, HES-, and HSH-treated groups. (L: lumen; MC: mesangial cell; RC: red cell; WC: white cell). ^△^ *P* ≤ 0.05 compared to group C; ^×^ *P* ≤ 0.05 compared to group S; ^▲^ *P* ≤ 0.05 compared to the HTS group.

Small-volume resuscitation with HES and HSH markedly decreased the augmented Scr and BUN levels (*P* = 0.028 versus group S) due to LPS exposure, while HTS resuscitation was less effective (Figures 1(a) and 1(b)).

LPS caused severe renal tubular injury, manifested as significant tubular degeneration, disintegration, edema, necrosis, and abscission of renal tubular epithelium, as well as cellular debris obstructing the collecting tubules and basal membrane fracture (Figures 1(d) and 1(e)). Small-volume resuscitation with colloid and hyperoncotic solutions markedly ameliorated the renal injury, showing that local epithelial and mitochondrial edema, cellular necrosis, and abscission were seen in the HTS group, while kidneys in the HES and HSH groups appeared with only slight edema with occasional cellular necrosis and abscission. These changes were correlated with the Paller histology grading of LPS-mediated kidney damage at 60 min after resuscitation (Figure 1(c)).

### 3.2. Effects of Small-Volume Resuscitation on Systemic Circulation and Renal Perfusion

SAP was measured to evaluate the systemic circulatory state and renal perfusion was detected through Doppler ultrasound by simultaneously monitoring renal blood flow signals (CVI), *V*_max⁡_, *V*_min⁡_, and the calculated RVRI [20]. Based on the measurement of these parameters, the LPS-induced deterioration of systemic and renal hemodynamics was assessed by comparisons among the five groups at the *T*_0_ time point, while improvement after small-volume resuscitation was evaluated by comparisons among the five groups at the *T*_1_ time point.

**Figure 2:**
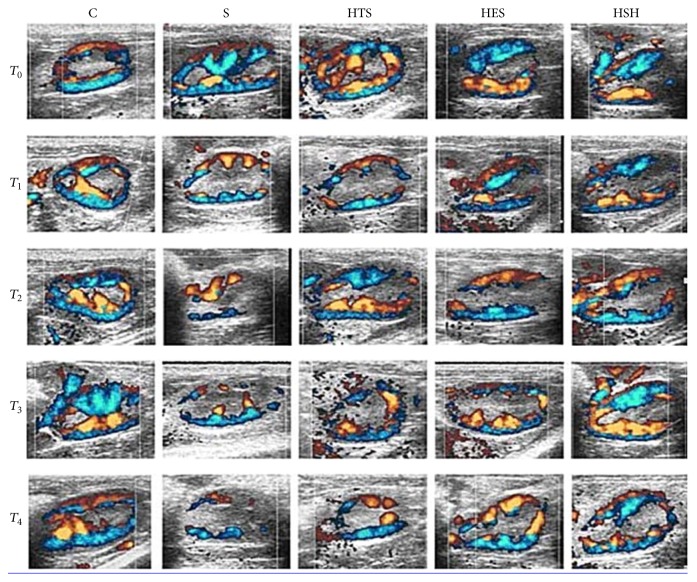
Color velocity imaging (CVI) of kidney at left renal door cross-sectional level. In group C and other groups at the *T*_0_ time point, renal blood flow signals were strongly and uniformly distributed with adequate cortical perfusion, while it decreased dramatically with discrepant distribution and weak cortical perfusion at *T*_1_ in the four experimental groups, among which there existed some degree of improvement with time in the HTS, HES, and HSH groups.

Doppler ultrasound showed that renal blood flow signal (CVI) was strongly and uniformly distributed with adequate cortex perfusion before LPS administration and then dramatically decreased with discrepant distribution and weak renal perfusion (CVI) under LPS challenge. Small-volume resuscitation with HES and HSH markedly improved CVI, while saline and HTS showed only slight improvement on renal blood flow (Figure 2).

**Figure 3:**
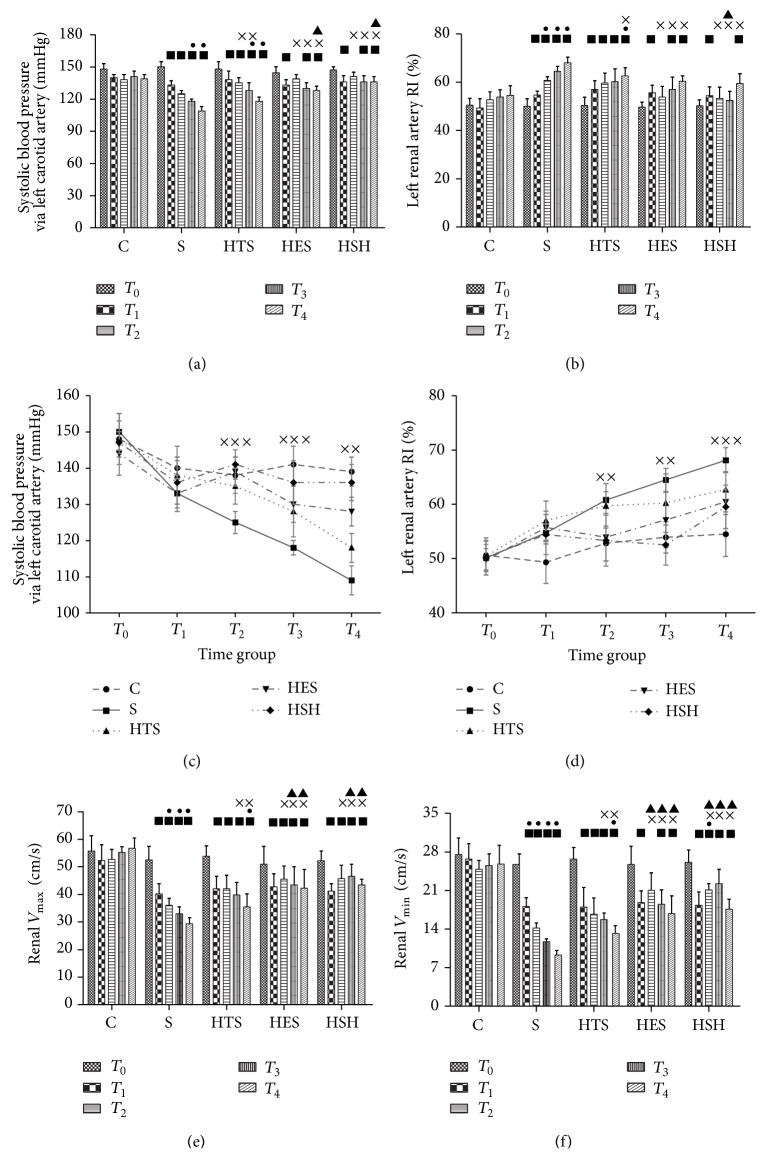
Small-volume resuscitation improved systemic and renal hemodynamics (mean ± SEM, *n* = 6). (a), (b), (e), and (f) show the early stage alterations to SAP, RVRI, *V*_max⁡_, and *V*_min⁡_ in different groups over time. ^■^ *P* ≤ 0.05 versus *T*_0_; ^●^ *P* ≤ 0.05 versus *T*_1_; ^×^ *P* ≤ 0.05 versus group S; ^▲^ *P* ≤ 0.05 versus group HTS. (c) and (d) are the line charts for mean arterial blood pressure (MAP) expressed in mmHg throughout the experimental procedure and left renal artery RI (RARI) expressed as (*V*_max⁡_ − *V*_min⁡_)/*V*_max⁡_. ^×××^ *P* ≤ 0.05, S versus HTS, HES, and HSH; ^××^ *P* ≤ 0.05, S versus HES and HSH.

The time courses for measurements of SAP, *V*_max⁡_, *V*_min⁡_, RVRI, and CVI throughout the five sequential study time points are presented in Figures 2 and 3. LPS caused marked decreases in SAP, *V*_max⁡_, and *V*_min⁡_, and increased RVRI (*P* = 0.012 versus *T*_0_); effects of LPS continuously worsened over time in group S (*P* = 0.028 versus *T*_0_ and *T*_1_). Although resuscitation with HES and HSH failed to completely restore systemic and renal hemodynamics to baseline (*P* = 0.017, versus *T*_0_), the detected associated parameters were immediately and persistently improved (*P* = 0.027, versus *T*_1_), while HTS only temporarily ameliorated these indices (*P* = 0.039; *T*_2_ versus *T*_1_). Figures 3(c) and 3(d) (line graph) illustrate date shown in Figures 3(a) and 3(b) (histogram) in a different manner.

### 3.3. Effects of Small-Volume Resuscitation on Renal Cortical TNF-*α* and NO Levels

**Figure 4:**
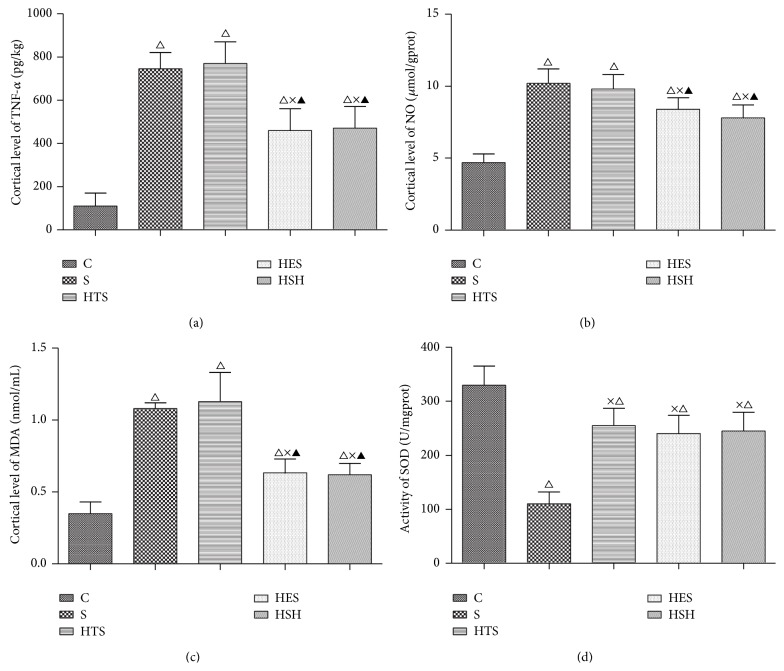
Variations in biochemical indexes in the different treatment groups. ^△^ *P* ≤ 0.0, compared to group C; ^×^ *P* ≤ 0.05 compared to group S; ^▲^ *P* ≤ 0.05 compared to the HTS group.

Production of TNF-*α* and production of NO, two important inflammatory mediators, were both promoted by the LPS treatment, demonstrating generation of an inflammatory response. In this study, the levels of renal cortical TNF-*α* and NO markedly increased after LPS challenge (*P* = 0.001; all versus group C) and were influenced to different degrees by the four types of fluid resuscitation. In the HES and HSH groups, TNF-*α* levels decreased (*P* = 0.013 versus group S) (Figure 4(a)) and NO levels did not change, while in the HTS group both TNF-*α* and NO were maintained at high levels (Figure 4(b)).

### 3.4. Effects of Small-Volume Resuscitation on Renal Cortical MDA Level and SOD Activity

MDA, a by-product of lipid peroxidation, reflects the extent of oxidative damage, while the activity of SOD, a critical antioxidant, can be used to represent the antioxidative capacity. LPS challenge increased renal cortical levels of MDA and inhibited activity of SOD (*P* = 0.002; all versus group C). Small-volume resuscitation improved the renal oxidative stress state, with resuscitation with HES and HSH decreasing the augmented MDA level and enhancing the SOD activity (*P* = 0.036, versus group S or HTS) (Figures 4(c) and 4(d)).

## 4. Discussion

Severe infection causes endotoxemia and the kidney is one of the first organ systems affected by LPS [21]. Reports indicate that endotoxemia-induced renal hypoperfusion contributes to an ischemia-reperfusion insult that potentially leads to the activation of renal inflammation and oxidative stress [10]. Fluid resuscitation has always been recognized as the primary resuscitation strategy for patients with AKI after endotoxemia [7, 11, 12, 20]. Although large amounts of fluid infusion could quickly improve renal perfusion, it is strictly limited to use in some critically ill patients suffering from endotoxemia and is contraindicated in patients with heart dysfunctions [22].

Small-volume resuscitation with hypertonic fluids has been regarded as an effective strategy for hemorrhagic shock, but it is unclear how different kinds of fluids affect kidney injury induced by LPS. Hence, our purpose here was to investigate the varying ameliorating effects of small-volume resuscitation with saline, 7.5% hypertonic saline, hydroxyethyl starch 130/0.4, and hypertonic saline hydroxyethyl starch 40 on LPS-induced AKI. Based on the available reports [23], we used the intravenous resuscitation dose of 4 mL/kg as an effective and safe dose. To this end, we conducted assessments of pathology images, systemic blood pressure, and renal perfusion, as well as renal levels of TNF-*α*, NO, and MDA and activity of SOD. Our results showed that, as compared with saline, small-volume hydroxyethyl starch 130/0.4 and hypertonic saline hydroxyethyl starch 40 could rapidly ameliorate the severity or postpone the development of renal tubular injury in the rat model of endotoxemia. At the same time, hydroxyethyl starch 130/0.4 and hypertonic saline hydroxyethyl starch 40 immediately restored systemic and renal blood flow and even reduced the renal inflammatory response and oxidative stress. In contrast, 7.5% hypertonic saline only temporarily improved systemic circulation and renal blood flow without having effects on cytokine release.

Endotoxemia has been proven to be associated with progressive renal dysfunction, even in the presence of normal or elevated blood pressure and cardiac output [13, 14]. Insight into renal microcirculation should provide more meaningful information about the underlying mechanisms of renal dysfunction. Doppler ultrasound is a precise method to evaluate renal blood flow [24]. In the present study, we applied Doppler ultrasound and showed that small-volume resuscitation with HES and HSH immediately and permanently improved the deceased renal blood flow that resulted from LPS challenge, while HTS showed only slight and temporary effects compared to those elicited by saline. Meanwhile, parameters for systemic blood pressure evolved in coincidence with the trend of renal blood flow and pathological damage. These results suggest that the choice of different fluids is closely related to the improvement by small-volume resuscitation of renal perfusion. Accordingly, we conclude that infusion of colloids is better for clinical small-volume resuscitation for endotoxemia patients having complications with heart dysfunction. Furthermore, because the small-volume resuscitation could not completely restore systemic circulation and renal blood flow, other strategies should be adopted to improve the protective effect, including resuscitation in combination with vasoactive or organ-protective drugs.

Normal saline, an isotonic solution, is rapidly distributed around vessels, while hypertonic saline can transfer interstitial fluids into vessels to improve circulation based on its hypertonicity. However, it has a short duration of action because of the rapid reestablishment of osmotic balance between intra- and extracellular fluids. HES and HSH can elevate colloid osmotic pressure and stay longer in vessels to effectively maintain intravascular volume [25]. This may be the main reason why small-volume resuscitation with HES and HSH alleviates kidney injury by improving renal hemodynamics and microperfusion.

During the initial period of endotoxemia, it is the severity of the inflammatory response and oxidative stress that trigger microvascular dysfunction and early organ failure, and treatment during the first six to twenty-four hours is critical for patient outcome and survival [26–28]. In addition to cytokine storms (e.g., TNF-*α*, IFN-*γ*, and IL), NO has been increasingly recognized as a biological mediator that plays an important role in the damage process [29]. As to sepsis, both experimental and clinical investigations have revealed enhanced NO in the plasma, which correlates inversely with arterial BP [30]. Here, we analyzed alterations to the levels of TNF-*α*, NO, and MDA, as well as SOD activity, and found that small-volume resuscitation with HSH and HES reduced renal cortical inflammatory response and oxidative stress, while HTS did not. Several studies of fluid resuscitation have also verified that renal perfusion is closely related to local renal oxidative and inflammatory response in endotoxic and hemorrhagic shock models [31]. Based on the previous and these current results, we conclude that HSH and HES were more effective in improving renal blood flow. This was associated with decreases in renal inflammatory response and oxidative stress.

Considerable controversy exists, however, about the safety and effectiveness of using colloid solutions in critical care units. Of note, one clinical trial indicated that application of HES is associated with increased rates of renal-replacement therapy [32]. Indeed, patients in this trial repeatedly or predominantly received infusion with HES, resulting in collagen deposition in the perinephric space, contributing to the main cause of HES-associated renal injury. Similarly, another randomized controlled study found that HES provided significantly better lactate clearance and less renal injury than saline [2]. The authors concluded that resuscitation with hydroxyethyl starch improves renal function and lactate clearance in penetrating trauma [33]. In the present study, we showed that early single resuscitation with small-volume colloids or hyperoncotic solutions could effectively alleviate renal injury and was better than the use of crystalloids. Therefore, our results provide a rationale and valuable strategy for ameliorating renal injury in the early stage of endotoxemia using resuscitation with a single small volume of colloids or hyperoncotic solutions, especially for patients complicated with heart dysfunction.

Indeed, we have several limitations in this study. The first is that we did not assess the long-term outcome of rats, including survival rate and mortality rate observation. Secondly, albumin is another kind of colloids being considered to infuse during the early resuscitation of patients with severe sepsis. In the future study, we will replenish the detected parameters and added group albumin to make the investigation more integrated.

In conclusion, our study provides the important finding that early small-volume resuscitation with single small volumes of hydroxyethyl starch 130/0.4 and hypertonic saline hydroxyethyl starch 40 leads to renal-protective effects from the damage due to LPS exposure.
